# BI-HEX^®^-GlymaxX^® ^cells enable efficient production of next generation biomolecules with enhanced ADCC activity

**DOI:** 10.1186/1753-6561-7-S6-P63

**Published:** 2013-12-04

**Authors:** Anja Puklowski, Till Wenger, Simone Schatz, Jennifer Koenitzer, Jochen Schaub, Barbara Enenkel, Anurag Khetan, Hitto Kaufmann, Anne B Tolstrup

**Affiliations:** 1 Boehringer-Ingelheim, Biberach an der Riss, Germany, 88397

## Background

Despite the succes story of therapeutic monoclonal antibodies (mAbs), a medical need remains to improve their efficacy. One possibility to achieve this is to modulate important effector functions such as the antibody dependent cellular cytotoxicity (ADCC). The advantage of highly active biotherapeutic molecules is - apart from the enhanced efficacy - the reduction of side effects due to lower administered doses. Furthermore, these therapeutic antibodies may enable treatment of current non-responders, e.g. patients with low antigen bearing tumors. Enhancement of the effector functions of antibodies can be achieved either by directly mutating the antibody's amino acid sequence or by modifying its glycosylation pattern, e.g. by using a novel host cell line able to attach a desired glycostructure to the product. The latter approach has the advantage of not impacting the antibody structure itself, thereby avoiding negative effects on the PK/PD of the molecule. During the last decade it has been shown that antibodies with a reduced level of glycan fucosylation are much more potent in mediating ADCC, a mode of action particularly relevant for cancer therapeutics. Therefore, defucosylated antibodies are of major interest for biotherapeutics developers. To produce such antibodies, Boehringer Ingelheim has inlicensed the GlymaxX^® ^system from ProBioGen, Germany. This technology utilises the bacterial protein RMD (GDP-6-deoxy-D-lyxo-4-hexulose reductase) which, when stably integrated into host cell lines, inhibits fucose de-novo biosynthesis. The enzyme deflects the fucosylation pathway by turning an intermediate (GDP-4-Keto-6-Deoxymannose) into GDP-Rhamnose, a sugar that cannot be metabolised by CHO cells. As a consequence, recombinant antibodies generated by such host cells exhibit reduced glycan fucosylation and 20-100 fold higher ADCC activity. Here, we show the establishment of a new host cell line, termed BI-HEX^®^-GlymaxX^® ^which is capable of producing highly active therapeutic antibodies. We furthermore present data on the cell line properties concerning cell culture performance (e.g. titer, growth, transfection efficiency), process robustness and product quality reproducibility.

## Methods

The BI-HEX^® ^host cell line was transfected with the bacterial RMD enzyme and stably expressing clones were selected. The presence of RMD was confirmed by Western blotting. The clones were analysed for stability of RMD expression over time in continous culture (>100 days), glycoprofile structure, CD16 binding and ADCC activity of mAbs produced by these clones before selection of the final new BI-HEX^®^-GlymaxX^® ^host cell. Furthermore, we examined the growth and cultivation properties of the modified BI-HEX®GlymaxX^® ^cells to ensure that the engineered host cell maintained the favourable manufacturability properties of BI-HEX^® ^and we tested the reproducibility of key product quality attributes of the generated antibodies.

## Results

**Figure 1:**
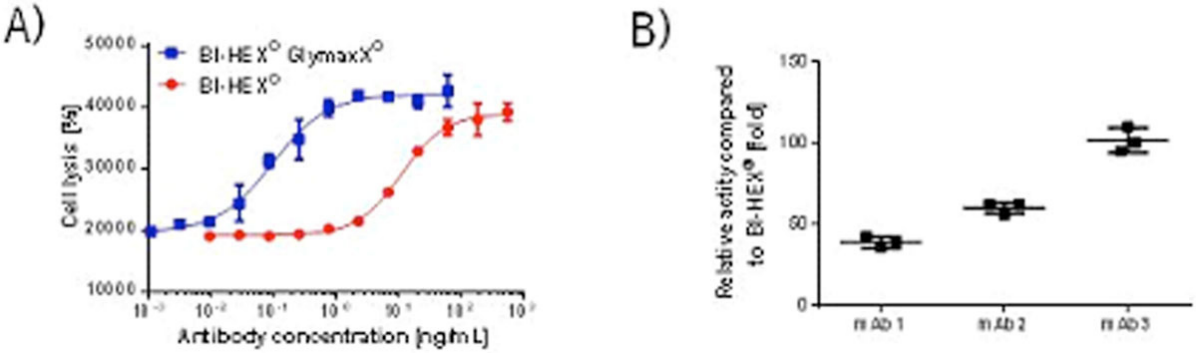
**A) Comparison of ADCC activity of Rituximab produced in either BI-HEX^® ^or BI-HEX^®^-GlymaxX^® ^**. **B) **ADCC activity of 3 different mAbs produced in BI-HEX^®^-GlymaxX^® ^. Three independent production runs were performed for each mAb. The mAbs were individually purified by protein A capture before ADCC activity determination.

Up to date seven different antibodies were produced in our new BI-HEX^®^-GlymaxX^®^host cell line. All molecules showed a very significant reduction of fucosylation down to 1-3% compared to the control. Correlating with the low fucose levels, antibodies produced in BI-HEX^®^-GlymaxX^® ^exhibited a 20-100× increased ADCC activity (Figure 1A). This enhancement also correlated well with an increase in CD16 binding. For the routine cell line and process development we investigated the robustness of the defucosylation and its resulting activity enhancement. The results indicated a high reproducibility between independent production runs. The ADCC level as well as the CD16 binding was robust for all analysed mAbs (Figure 1B). Investigating the cell culture behaviour of the BI-HEX^®^-GlymaxX^®^and its parental BI-HEX^® ^cell line, we saw comparable results for their transfection efficiencies, doubling times, titer and production run performance. Depletion studies of RMD showed that this enzyme can be efficiently depleted during downstream purification of the mAb.

## Conclusions

Our new BI-HEX^®^-GlymaxX^®^cell line is capable of producing >90% defucosylated antibodies which exhibit a 20-100 fold higher ADCC activity compared to a normal CHO production cell line like BI-HEX^®^. This increase in ADCC activity correlated with a stronger CD16 binding in those molecules. Furthermore, the BI-HEX^®^-GlymaxX^® ^cells show the same manufacturing properties (transfection efficiency, doubling times, titer, peak cell density) to its originator cell line. For the depletion of RMD we've established a sensitive depletion assay and measured a complete reduction of RMD after the first purification step (protein A capture).

